# Source attribution of human *Campylobacter* infection: a multi-country model in the European Union

**DOI:** 10.3389/fmicb.2025.1519189

**Published:** 2025-02-05

**Authors:** Cecilie Thystrup, Maja Lykke Brinch, Clementine Henri, Lapo Mughini-Gras, Eelco Franz, Kinga Wieczorek, Montserrat Gutierrez, Deirdre M. Prendergast, Geraldine Duffy, Catherine M. Burgess, Declan Bolton, Julio Alvarez, Vicente Lopez-Chavarrias, Thomas Rosendal, Lurdes Clemente, Ana Amaro, Aldert L. Zomer, Katrine Grimstrup Joensen, Eva Møller Nielsen, Gaia Scavia, Magdalena Skarżyńska, Miguel Pinto, Mónica Oleastro, Wonhee Cha, Amandine Thépault, Katell Rivoal, Martine Denis, Marianne Chemaly, Tine Hald

**Affiliations:** ^1^National Food Institute, Technical University of Denmark, Lyngby, Denmark; ^2^National Institute for Public Health and the Environment (RIVM), Bilthoven, Netherlands; ^3^Institute for Risk Assessment Sciences, Utrecht University, Utrecht, Netherlands; ^4^Department of Food Safety, NVRI, Pulawy, Poland; ^5^Food Microbiology, Department of Agriculture, Food and the Marine, Celbridge, Ireland; ^6^Teagasc Food Research Centre, Dublin, Ireland; ^7^VISAVET Health Surveillance Center, Universidad Complutense, Madrid, Spain; ^8^Animal Health Department, Faculty of Veterinary, Universidad Complutense, Madrid, Spain; ^9^Epidemiology, Surveillance and Risk Assessment, Swedish Veterinary Agency, Uppsala, Sweden; ^10^National Institute of Agrarian and Veterinary Research, (INIAV), Oeiras, Portugal; ^11^Division of Infectious Diseases and Immunology, Faculty of Veterinary Medicine, Utrecht University, Utrecht, Netherlands; ^12^Department of Bacteria, Parasites and Fungi, Statens Serum Institut, Copenhagen, Denmark; ^13^Department of Food Safety, Nutrition and Veterinary Public Health, Istituto Superiore di Sanitá, Rome, Italy; ^14^Department of Microbiology, National Veterinary Research Institute (PIWet), Pulawy, Poland; ^15^Department of Infectious Diseases, National Institute of Health Doutor Ricardo Jorge (INSA), Lisbon, Portugal; ^16^Unit of Hygiene and Quality of Poultry and Pork Products, Laboratory of Ploufragan-Plouzané-Niort, French Agency for Food Environmental and Occupational Health and Safety (ANSES), Ploufragan, France

**Keywords:** source attribution, foodborne disease, campylobacteriosis, machine learning, European union

## Abstract

**Introduction:**

Infections caused by *Campylobacter* spp. represent a severe threat to public health worldwide. National action plans have included source attribution studies as a way to quantify the contribution of specific sources and understand the dynamic of transmission of foodborne pathogens like *Salmonella* and *Campylobacter*. Such information is crucial for implementing targeted intervention. The aim of this study was to predict the sources of human campylobacteriosis cases across multiple countries using available whole-genome sequencing (WGS) data and explore the impact of data availability and sample size distribution in a multi-country source attribution model.

**Methods:**

We constructed a machine-learning model using *k*-mer frequency patterns as input data to predict human campylobacteriosis cases per source. We then constructed a multi-country model based on data from all countries. Results using different sampling strategies were compared to assess the impact of unbalanced datasets on the prediction of the cases.

**Results:**

The results showed that the variety of sources sampled and the quantity of samples from each source impacted the performance of the model. Most cases were attributed to broilers or cattle for the individual and multi-country models. The proportion of cases that could be attributed with 70% probability to a source decreased when using the down-sampled data set (535 vs. 273 of 2627 cases). The baseline model showed a higher sensitivity compared to the down-sampled model, where samples per source were more evenly distributed. The proportion of cases attributed to non-domestic source was higher but varied depending on the sampling strategy. Both models showed that most cases could be attributed to domestic sources in each country (baseline: 248/273 cases, 91%; down-sampled: 361/535 cases, 67%;).

**Discussion:**

The sample sizes per source and the variety of sources included in the model influence the accuracy of the model and consequently the uncertainty of the predicted estimates. The attribution estimates for sources with a high number of samples available tend to be overestimated, whereas the estimates for source with only a few samples tend to be underestimated. Reccomendations for future sampling strategies include to aim for a more balanced sample distribution to improve the overall accuracy and utility of source attribution efforts.

## Introduction

Infections caused by *Campylobacter* spp. represent a severe threat to public health worldwide. This foodborne pathogen, known for its high prevalence in food production animals like poultry, cattle, and pigs, is mainly transmitted through contaminated undercooked meat and raw milk (World Health Organization, 2024). In the European Union (EU), *Campylobacter* alone was responsible for more than 137,000 confirmed human cases of campylobacteriosis in 2022 (European Food Safety Authority, European Centre for Disease Prevention and Control, 2023), which highlights the need for targeted intervention methods to reduce further cases. In the European Union (EU), *Campylobacter* spp. is considered a zoonotic agent of priority with mandatory monitoring.

In the current approach to managing food-related diseases, an emphasis is placed on surveillance and monitoring. Sampling is conducted to monitor and track disease trends and is typically collected through national monitoring programs (NMPs) or other projects (Gaia et al., 2021). As a result, most samples are usually collected from food and animal sources believed to contribute most to human infections, meaning that the available data is skewed toward the selected sources (Lassen et al., 2024). This can impact the representativeness of the monitoring data available for source attribution analyses. Source attribution models link sporadic human cases of an illness to different sources like food and animal reservoirs (Munck et al., 2020). These models can predict the probability of human cases originating from a particular food or animal reservoir, providing information about the most important sources that stakeholders and policymakers can use to establish interventions to control or prevent transmission routes and ultimately reduce the number of human infections. In Denmark, national action plans have included source attribution as a way to understand the transmission pathways of different pathogens in the food production chain (Channie Kahl Petersen, 2021). As a result of this, *Salmonella* spp. has been reduced effectively in the broiler and table egg production in Denmark (Danish Veterinary and Food Administration, 2025; Wegener et al., 2003). Both *Salmonella* and *Campylobacter* is still being managed by action plans, which continues to use source attribution to inform risk management (Lassen et al., 2024).

Numerous source attribution models have been developed in recent years. In Denmark, source attribution has routinely been performed using the Hald model (Hald et al., 2004), which has been modified and subsequently adopted by other countries like Australia, New Zealand, and the Netherlands (Mullner et al., 2009; Glass et al., 2016; Fearnley et al., 2018; Mughini-Gras et al., 2021). The development of machine-learning algorithms that use supervised classification models to predict the probability of a case originating from a specific source has paved the way for new methods (Munck et al., 2020). Combined with the use of WGS data, these approaches have become the preference for characterizing bacterial isolates from food and animal products in source attribution (Arning et al., 2021).

With the use of WGS data, source attribution models can infer allelic variations between sources and accurately predict the origin of a specific isolate causing infection (Munck et al., 2020). Both core genome multi-locus sequence typing (cgMLST) and *k*-merisation have been used for taxonomic analysis in numerous studies and have proved to be quite efficient, especially for large genomes (Bernard et al., 2016; Panyukov et al., 2020). Based on the use of short oligonucleotides and free from alignment, *k*-mer counting can be used to determine the number of *k*-mers matching a reference sequence, either against a reference genome or against other genomes of interest (Zielezinski et al., 2017). The idea of using *k*-mers to distinguish between different genomes of *Campylobacter* is coupled to the concept of genomic signatures, which was first introduced for dinucleotide composition (e.g., GC content) by Deschavanne et al. during 1999. In this case, the assumption is that *Campylobacter* infections from the same source share a similar frequency of *k-*mers, thereby making it possible to track infections across different reservoirs (Deschavanne et al., 1999).

Most source attribution models are focused on transmission within single countries and do not account for cross-border contamination in imported food. In Denmark alone, while approximately 140,000 tons of meat products were imported in 2023, the country exported more than 1 million tons to other countries (Statistics Denmark, 2024). This trade with animal-food products between member states can significantly exacerbate the spread of diseases like campylobacteriosis, especially in the EU, where food products have no barriers to trade.

This study aimed to attribute human sporadic and domestically acquired *Campylobacter* infections to sources across multiple countries using data from seven EU member states. We modified the source attribution models developed by Munck et al. (2020) and Brinch et al. (2023) to explore the impact of data availability and sample distribution on the multi-country source attribution estimates and compared these with the results from the individual country models.

## Materials and methods

### Data collection and pre-processing

Existing surveillance and monitoring data were collected as part of the EU project “Discovering the sources of *Salmonella*, *Campylobacter*, VTEC and antimicrobial resistance” (DiSCoVer) project.^1^ WGS data of *Campylobacter* spp. from eight countries (Denmark, France, Ireland, the Netherlands, Poland, Portugal, Spain, and Sweden) were used. The data set included isolates from humans, the environment, such as freshwater and wastewater source, and a variety of animal and food sources, such as chicken, ruminants and other animals (Table 1). The sampling strategies varied across countries, with differences in both the types of sources sampled and the number of samples collected from each source.

**Table 1:** Number of *Campylobacter* spp. isolates from each animal and food source and humans included in the model.

	DK	ES	FR	IE	NL	PL	PT	SE
Broiler	829	114	61	272	254	162	94	50
Broiler^i^	111	–	–	–	–	–	10	–
Cat	–	–	4	–	15	–	3	–
Cattle	253	46	39	24	207	6	–	–
Dog	21	–	27	–	85	–	15	–
Duck	4	–	–	–	–	–	–	–
Duck^i^	22	–	–	–	–	–	–	–
Freshwater	2	–	28	–	59	–	27	–
Pig	27	17	10	6	110	77	7	–
Sheep/goat	–	–	–	5	110	–	–	–
Turkey	–	28	–	–	38	–	16	–
Turkey^i^	9	–	–	–	–	–	–	–
Wastewater	10	21	–	–	194	–	–	–
Wild animals	–	14	2	–	–	–	3	–
Wild bird	–	7	21	–	63	–	7	40
Human	1,558	128	–	267	280	15	379	–

^iImported sources. DK, Denmark; ES, Spain; FR, France; IE, Ireland; NL, the Netherlands; PL, Poland; PT, Portugal; SE, Sweden.^

The individual countries provided metadata, while sequences were either downloaded from European Nucleotide Archive (ENA) or provided directly by the involved countries. Metadata included information on the country of origin, the institute responsible for the sampling, the year of collection, and the sampling material (manure/faces, fresh meat, carcass swab, etc.). For humans, information on whether the case was domestic or travel-related, or part of an outbreak or sporadic, was also noted. Samples with missing metadata were removed from the data set. Human cases with a travel history or cases related to outbreaks were removed to focus the model on sporadic and domestically acquired cases. The sample sources were grouped based on the reservoir in order to reduce the number of different groups of sources (for example, layer chicken and broilers were collapsed into a single category).

The WGS data were pre-processed as part of the in-house Food QC-and assembly pipeline, using bbduk (Bushnell et al., 2017) and FastQC (Simon, 2024) for adapter trimming and quality checking. Assembly of the trimmed genomes was performed using the assembler SPAdes (version 3.9) (Prjibelski et al., 2020). Median number of contigs was 29 (range 1–4,305) and N50 ranged between 560 and 1,105,278 (median: 160,175).

### *K*-mer counting

Assembled genomes were used for *k*-mer extraction using the tool KMC (version 3.0) (Kokot et al., 2017) with length of *k* = 9. An in-house Python script was used to combine the *k*-mer frequencies from all samples into a single matrix, where each row corresponded to a sample and each column represented an individual *k*-mer. For each *k*-mer, its standard deviation across all samples were calculated based on its frequency values using the formula for standard deviations for samples. For example, if a *k*-mer had frequencies of 15, 10, and 22 across three samples, its standard deviation was calculated as 6.03.

To reduce the size of the matrix, any *k-*mers with a standard deviation of less than 10 were removed from the combined matrix.

### Machine learning

**Figure 1:**
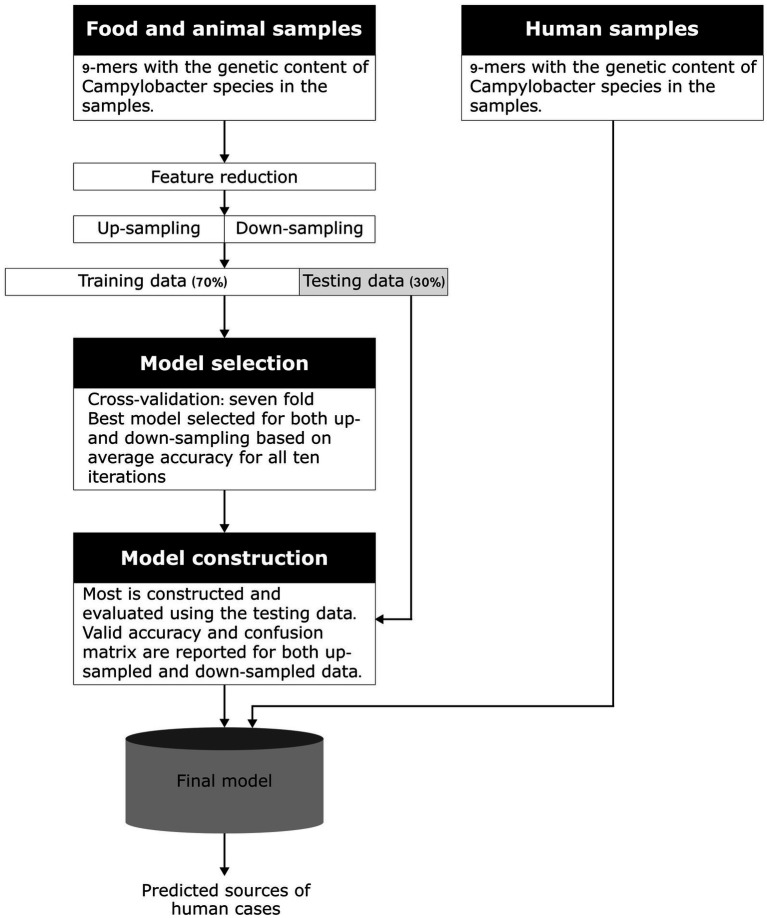
Conceptual model of the machine learning method using *k*-mers. Adapted from Munck et al. (2020).

A machine-learning model developed by Brinch et al. (2023) to predict the sources of human campylobacteriosis cases in Denmark using *k*-mers was adapted to this study. Click or tap here to enter text. First, models for each country with human WGS data available were constructed, which was followed by the construction of a multi-country model based on data from all the participating countries. A flowchart showing the modeling process is available in Figure 1.

### Feature reduction and up-sampling

Feature reduction was carried out on the matrix to reduce the number of 9*-*mers in the final model using the caret package (version 6.0–94) and the Boruta package (version 8.0.0). The near-zero-variance method was used to reduce the number of 9-mers. The Boruta algorithm, which uses the random forest classifier, was then applied to select important attributes in a given dataset.

Up-sampling was applied to balance the dataset by artificially increasing the number of samples in underrepresented source categories to match those in the largest category. This meant that sources with less than 50 isolates were up-sampled to the largest number of isolates. For example, ‘duck’ was up-sampled from 4 to 829 in the Danish model. The up-sampled model was denoted as the baseline model.

To further investigate the impact of sampling on the model’s performance, down-sampling was also performed to reduce the number of samples in overrepresented categories to match the size of a smaller category (Kuhn and Johnson, 2013). Sources exceeding 300 samples were down-sampled to achieve balance. For example, ‘broilers’ were reduced from 829 to 300 samples in the Danish model. Down-sampling avoids artificially inflating the number of samples as is done with up-sampling. The two strategies were then compared.

### Model selection

Two machine-learning algorithms, logit-boost, and random forest, which had previously been applied in sequencing studies successfully were evaluated (Ogutu et al., 2011; Machado et al., 2015; Njage et al., 2019, 2019; Brinch et al., 2023).

For the model selection, the source data were split into test-and training data. The training data was used to randomly generate training data sets corresponding to 70% of the total number of samples. The remaining 30% of the samples were used to evaluate the performance of the model using 7-fold cross-validation. After 10 iterations, the accuracy of each algorithm was assessed, and the algorithm with the highest accuracy was selected for model construction.

### Model construction and evaluation

The selected model was constructed again following the modeling procedure as described in model selection. The model’s performance was evaluated using its valid accuracy, which is the accuracy of the cross-validation, kappa value, and confusion matrix. Cross-validation is a statistical method to assess the generalizability of the model. In this study, the dataset was split into multiple subsets, where the model was trained on some part of the data and tested on the remaining. This process was repeated several times to ensure robust performance and to reduce the risk of overfitting. The final accuracy metric reflected the models’ ability to predict the sources of the samples in the non-human data set. The confusion matrix further assessed the model’s ability to predict the sources of the samples. The confusion matrix compares the predicted classifications of the model to the true known classifications, while summarizing true positives, true negatives, false positives and false negatives. This allows for a more detailed understanding of the model’s performance by identifying, where predictions deviate from the observed, true sources (Kuhn, 2008). The kappa value is a measure that quantifies the agreement between observed and predicted sources. It was calculated by dividing the predicted number of sources minus the observed number of sources with the observed number of sources (Kuhn, 2008).

The proportion of samples predicted to the correct source (sensitivity) and the proportion of samples correctly predicted not to belong to a specific source (specificity) were also reported. The different performance measured were used to select the final model, which was then applied to determine the origin of the human campylobacteriosis cases by estimating the likelihood for each case to originate from a particular source.

## Results

The final dataset for the multi-country model included data from 6,313 *Campylobacter* isolates from humans with sporadic C*ampylobacter* infections (*n* = 2,627) and various sources (*n* = 3,686; Table 1). The number of samples and sources varied between countries. Sample sizes ranged from 90 to 2,846, with Denmark contributing the largest number of samples and Sweden the fewest. Similarly, the number of sources sampled per country varied from two to 10, with Denmark again sampling the most sources and Sweden the fewest. Denmark’s dataset included a substantial number of samples from imported meat. The dataset was also skewed toward broiler samples, particularly from Denmark (*n* = 829), Ireland (*n* = 272), and the Netherlands (*n* = 254), while other sources were underrepresented. France and Portugal sampled a relatively wide range of sources but collected fewer samples per source. Conversely, countries like Ireland and Poland sampled larger numbers of samples but from a more limited range of sources. A total of 431 human cases related to travel and outbreaks were excluded from the model. No human samples were available from France and Sweden.

The final *k*-mer matrix used in the source attribution model consisted of 3,996 9-mers after reduction. The Boruta algorithm found 277 confirmed 9-mers to be included in the multi-country model, based on the accuracies of the prediction of the source by each feature (9-mer).

### Individual model results

The performance measures of the random forest and logit-boost algorithm were compared for each country individually (Supplementary Table S1). The average accuracies, obtained from taking the average accuracy across 10 iterations, ranged from 0.507–0.959, with Spain having the lowest accuracy and Poland having the highest. The valid accuracy and the kappa-value for the selected algorithm were reported for each individual country model (Supplementary Table S1). The final model was run on individual cases using the model with the highest overall valid accuracy (Supplementary Table S1). The cumulative probability plot for each country showed that most cases could be attributed to domestically produced broilers (Supplementary Figures S1–S6). The total number of cases that could be attributed to each source were reported and the number of cases with at least a 0.7 probability to one source (Table 2). For all countries, this reduced the number of potential sources drastically, indicating that applying a threshold for the probability removes cases attributed with large uncertainty. The overall prediction of human cases across all countries showed that most could be attributed to broilers. Cattle and dogs were also found to be associated with human cases of campylobacteriosis.

**Table 2:** Results of the individual country models.

	DK	IE	NL	PL	PT	ES
Broiler	1,008 (731)	236 (236)	118 (64)	15 (15)	183 (102)	54 (36)
Broiler^i^	154 (16)	–	–	–	22 (0)	–
Cat	–	–	8 (0)	–	8 (0)	–
Cattle	296 (92)	24 (13)	51 (9)	0 (0)	–	32 (17)
Dog	36 (0)	–	29 (1)	–	44 (6)	–
Duck	3 (0)	–	–	–	–	–
Duck^i^	24 (0)	–	–	–	–	–
Freshwater	2 (0)	–	4 (0)	–	39 (10)	–
Pig	13 (4)	1 (0)	5 (2)	0 (0)	12 (0)	5 (1)
Sheep/goat	–	6 (4)	31 (1)	–	–	–
Turkey	–	–	14 (0)	–	48 (0)	13 (3)
Turkey^i^	20 (0)	–	–	–	–	–
Wastewater	3 (0)	–	16 (2)	–	–	16 (12)
Wild animals	–	–	–	–	7 (0)	7 (2)
Wild birds	–	–	5 (0)	–	16 (0)	1 (1)
Total number of cases	1,559 (843)	267 (253)	281 (79)	15 (15)	379 (118)	128 (72)

Each column shows the number of cases predicted for each specific source for that country. ^i^The numbers in parenthesis are Campylobacter infection cases with ≥70% probability of the case being attributed to that source. DK = Denmark, ES = Spain, IE = Ireland, NL = the Netherlands, PL = Poland, PT = Portugal.

### Selection and construction of multi-country model

The model selection showed the average accuracy using the baseline dataset was highest for the random forest algorithm (0.674, 95% CI 0.669–0.680) compared to the logit boost algorithm (0.666, 95% CI 0.658–0.673). When using the down-sampled dataset, the logit boost algorithm showed the highest average accuracy (0.67, 95% CI 0.6587–0.6818) compared to the random forest algorithm (0.654, 95% CI 0.645–0.663). The valid accuracy and the kappa value for the baseline and down-sampled models are shown in Supplementary Table S2. The average accuracies for both the baseline sampling strategy and the down-sampled strategy were similar, indicating that having a balanced dataset for the model selection did not improve the accuracy of the model. Therefore, the sensitivity and specificity of the different datasets were compared, using the model algorithm with the highest accuracy for each (baseline data: random-forest; down-sampled data: logit-boost).

For the down-sampled model, the specificity ranged from 0.97–1.0, indicating the model’s low misclassification rate of samples to incorrect sources (Supplementary Table S3). The sensitivity varied much more between sources, ranging from 0.07–1.0 with a single source having a sensitivity of 0.0, highlighting the model’s poor ability to predict sources correctly. The baseline model had a slightly lower specificity, ranging from 0.87–1.0, but had an overall higher sensitivity ranging from 0.2–1.0 (Supplementary Table S4), apart from a single source having a sensitivity of 0.0. Overall, due to the up-sampling, the sources with very few samples had a much higher sensitivity than sources with a higher number of samples. While the down-sampled model had a higher specificity (few samples allocated to the wrong source) overall, the much lower sensitivity suggested a shortfall in allocation samples to the correct source.

### Multi-country attribution model

**Figure 2:**
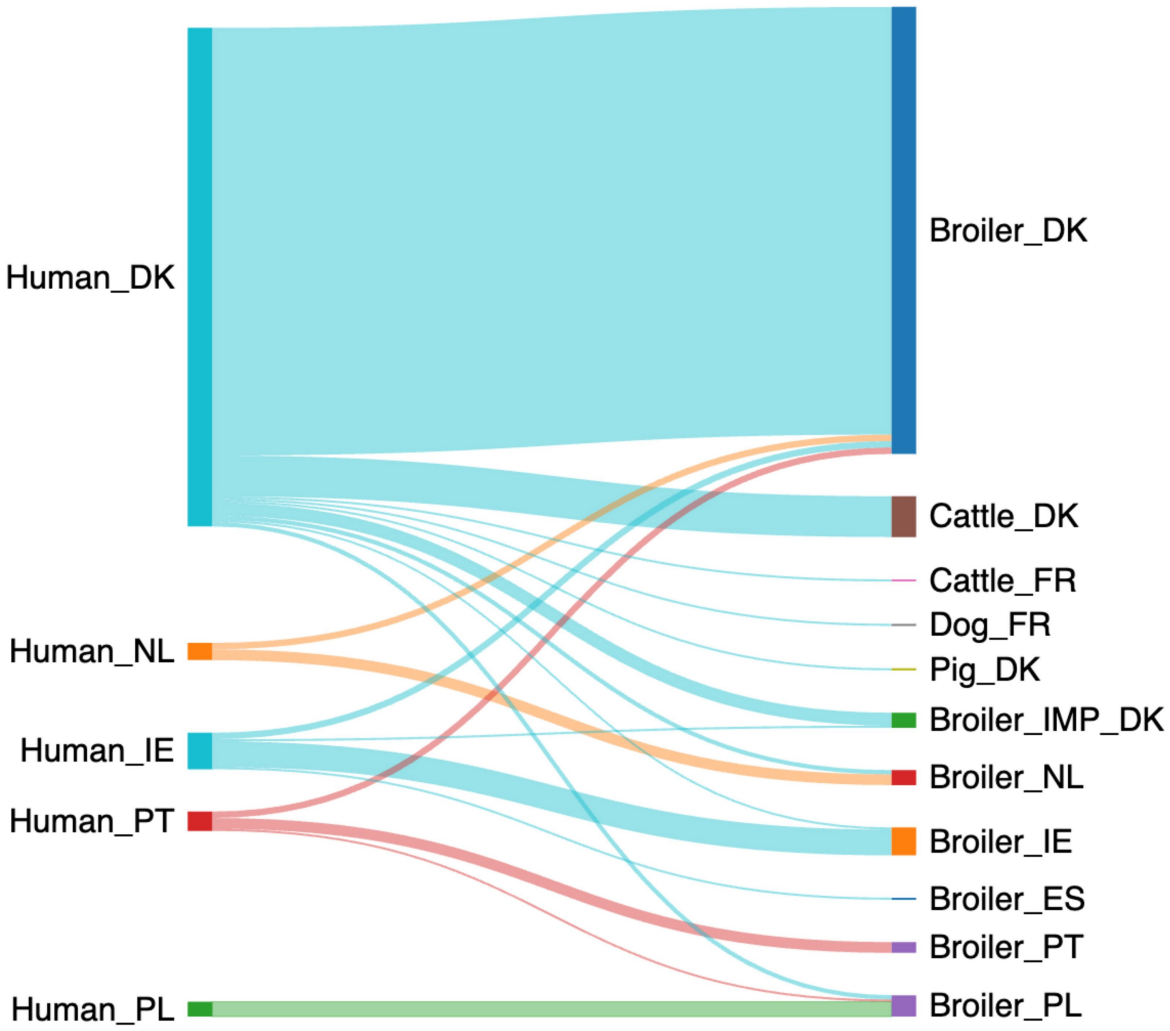
A Sankey diagram showing the probable (>70% probability) source of the 273 human campylobacteriosis cases using the baseline data (Made with sankeymatic.com). Numbers are available in Supplementary Table S5.

Using the baseline data in the logit boost algorithm, the machine-learning model predicted that 273 of the human campylobacteriosis cases could be attributed to a single source with at least 70% probability (Figure 2). According to the model, the majority of cases could be attributed to sources from the same country (248/273 cases, 91%), with fewer than 1 in 10 cases attributed to sources outside the country (Supplementary Table S5). Broilers from Denmark were the most important infection source for Danish cases (199/232 cases, 86%), followed by cattle from Denmark (19/232 cases, 8%). Few Danish cases could be attributed to sources outside of Denmark, like imported broiler meat (6/232 cases, 3%), meat from broilers in Poland (2/232 cases, 1%) or The Netherlands (2/232 cases, 1%). The cases from The Netherlands also showed a higher proportion of the infections attributed to broilers from The Netherlands (5/8 cases, 63%), with fewer attributed to Danish broilers (3/8 cases, 38%). The Portuguese cases were mainly attributed to broilers from Portugal (5/9 cases, 56%), with a few attributed to broilers from Denmark (3/9 cases, 33%) and a single case attributed to broilers in Poland (1/9 cases, 11%). For Ireland, the cases were mainly attributed to broilers from Ireland (12/16 cases, 71%) and a few were attributed to Danish broilers (3/17 cases, 18%). All the Polish samples were attributed to Polish broilers (7/7 cases, 100%).

**Figure 3:**
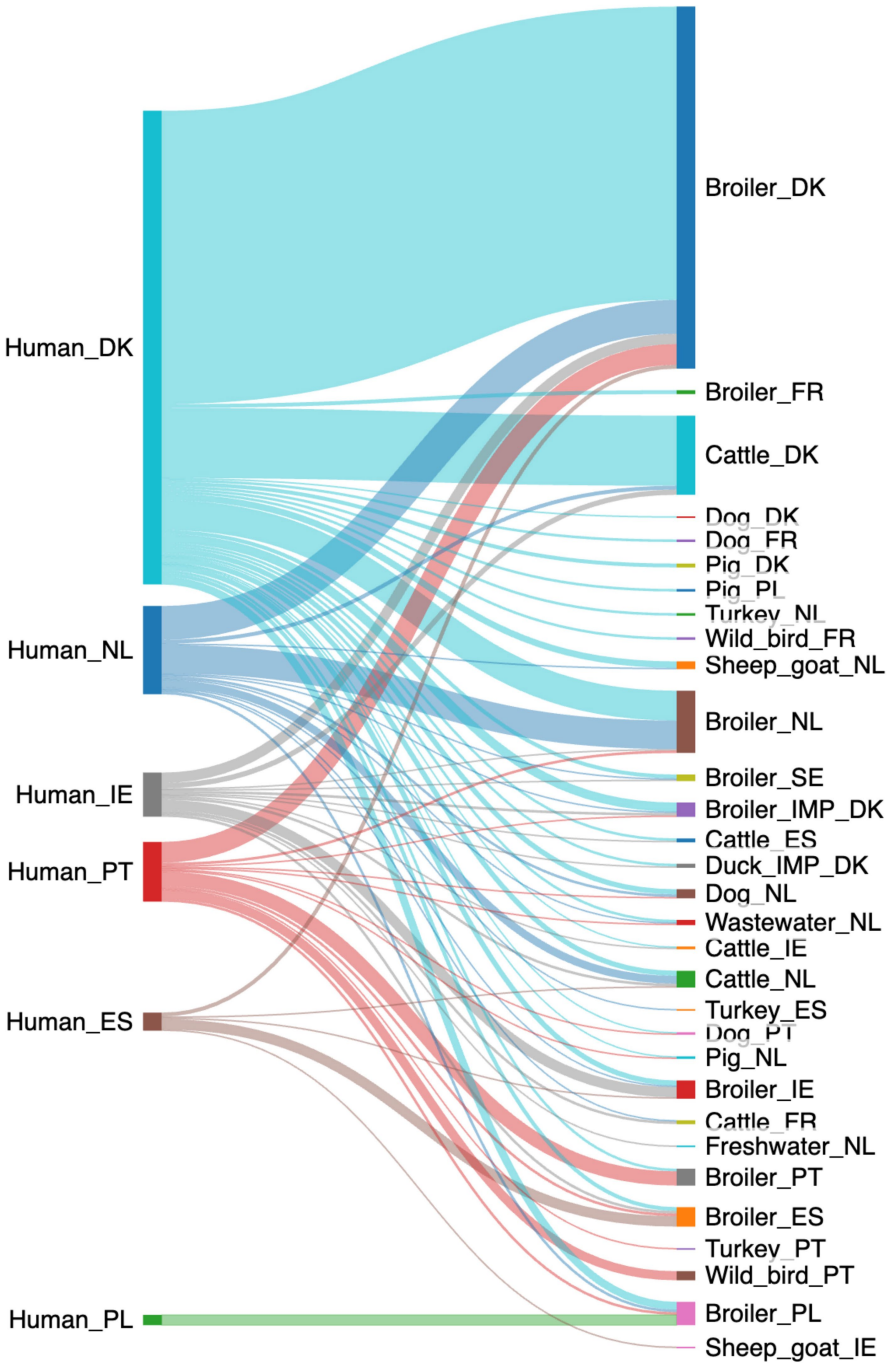
A Sankey diagram showing the probable (>70% probability) source of the 535 human campylobacteriosis cases using the down-sampled data (Made with sankeymatic.com). Numbers are available in Supplementary Table S6.

The machine-learning model using the down-sampled dataset with a random forest algorithm predicted 535 of the human campylobacteriosis cases with at least a 70% probability (Figure 3). Contrary to the baseline random forest model, the down-sampled logit boost model attributed 535 cases to a larger number of sources from 11 to 31 (Supplementary Table S6). Still, most cases could be attributed to domestic sources (361/535 cases, 67%), meaning that around 1 out of 3 cases (174/535 cases, 33%) could be attributed to sources from outside the country. Ireland had the largest proportion of cases attributed to non-domestic sources (23/34 cases, 68%). In contrast, Denmark had the lowest proportion of cases attributed to non-domestic sources (75/365 cases, 21%), except for Poland, where the sources were all predicted to be from Polish broilers.

As with the baseline model, cases from Denmark were mostly attributed to broilers from Denmark (226/365 cases, 62%), with some also attributed to cattle from Denmark (54/365 cases, 15%), as well as broilers from the Netherlands (22/365 cases, 6%). More cases from the Netherlands were attributed to Danish broilers (26/68 cases, 38%) than domestic broilers (22/68 cases, 32%). There was a similar trend for the cases from Portugal (16/46 cases attributed to Danish broilers, 35%) and Ireland (8/34 cases attributed to Danish broilers, 24%). Poland still had all cases attributed to domestic broilers (8/8, 100%), whereas Spain had the majority of cases attributed to domestic broilers (8/14 cases, 57%).

## Discussion

The aim of the study was to predict the relative contribution of sources to human Campylobacter infections using data from different European countries. By adapting the source attribution model introduced by Brinch et al. (2023), the effects of data availability and sampling distribution on the robustness and applicability of a multi-country source attribution model were assessed. The results showed that the distribution of sources, as well as the number of samples, had an impact on the sensitivity of the model. Applying the baseline source attribution model showed a higher sensitivity compared to the down-sampled model, where the sources were more evenly distributed. The notable difference suggested that the model’s ability to allocate samples to the correct source was better for the unevenly distributed dataset, even when one source was overrepresented.

The results from our multi-country modeling showed that while most cases could be attributed to domestic sources, there were still a notable likelihood that some cases originated from imported products. Imported food introduces additional complexity because genetic variability of *Campylobacter* strains in these products may differ significantly from each other and from the domestic strains, depending on the source of the sample (Stevens et al., 2024). To address this, one potential solution could be to introduce a weight matrix based on data regarding the proportion of specific food sources that are imported. Such a matrix would allow the model to assign different likelihood scores to attributions, reflecting the actual import volume of each source. For example, if a large proportion of a particular food type is imported, the model could adjust the probabilities accordingly to account for the higher likelihood of cross-border origins. However, the effectiveness of this approach would depend on the availability of data on food imports, which is not always accessible in all cases. Incorporating this information where available could significantly enhance the model’s capacity to account for cross-border food trade and improve the accuracy of source attribution.

Still, both models showed that the majority of cases could be attributed to sources domestic to each country. Most cases were attributed to broiler or cattle, both for the individual country models and the multi-country models, which correlates with numerous other studies on *Campylobacter* infections in humans (EFSA Panel on Biological Hazards, 2011; Cody et al., 2019; Douglas Inglis et al., 2021). Two studies from France and the Netherlands pointed to pets and environmental sources like wild birds and surface water as well, something that our results also support (Thépault et al., 2018; Mughini-Gras et al., 2021).

The analysis of the individual countries showed some disparities in the distribution of sources when implementing a threshold to the attribution of cases. A threshold of 0.7 was applied to the cases to ensure that only cases with a high likelihood of attribution to a given source were included in the final prediction. For some countries like Denmark and the Netherlands, this meant that when no threshold was implemented, the cases were distributed to a broader range of sources. The Netherlands included a large number of sources, but relatively few samples for each, which likely reduced the model’s ability to attribute cases with a high probability. So, when the threshold was imposed, this resulted in a pronounced narrowing of the range of sources to which human cases were attributed, suggesting a concentrated source profile within the country. In contrast, the data from Spain showed uniformity in the different distribution of sources, even when the threshold was applied. Despite the increased stringency of case attribution, the consistency in Spain’s source distribution indicates a more distributed source profile. This could likely be explained by the relatively few samples per source and a high number of sources, making it challenging for the model to discern clear patterns. In Portugal, a significant proportion of the samples were derived from bird-related sources (e.g., wild birds, turkey, broilers), which could have increased the genetic similarity among sources, making it more difficult to predict a source. For further source attribution modeling, this highlights the importance of choosing a suitable threshold. Implementing a threshold enhances the specificity and precision of the model but with the risk of losing important information about potential reservoirs.

When comparing the multi-country model’s valid accuracies with each individual country’s model, some individual country models had a lower accuracy than the multi-country model. This indicated that the model relied not only on the distribution and number of samples available from each source but also on the sample’s origin, suggesting that some sources increased the complexity of the source prediction. Wildlife reservoirs, such as wild birds, have been linked to human and animal cases of *Campylobacter* infection in other studies, although their exact role in their spread has not yet been established (Gardner et al., 2011; Cody et al., 2015). This variety in sources introduces complexity in the model, leading to lower precision, which is the case for Spain, the country with the lowest valid accuracy of 0.54 for the logit boost model.

The machine-learning model initially developed by Munck et al. (2020) was designed for cgMLST-based predictions. However, as highlighted in the study by Brinch et al. (2023), cgMLST data often suffer from the issue of missing alleles. To address this, imputation is typically required, but this process can introduce bias into the dataset. Such biases may influence the model’s ability to accurately capture the trends in the allele distributions, which leads to lower accuracy. The *k*-mer approach eliminates the issue of missing values entirely, as *k*-mers are directly extracted from the genomic sequences without relying on allelic information. Additionally, the *k*-mer-based model is computationally faster, which allows the model to run on larger datasets.

Limitations of this study include the variation in the number of samples available for each source, which highlights the need for a uniform sampling strategy when it comes to monitoring and surveillance of *Campylobacter* infections. Since sources was grouped based on shared animal reservoirs to increase the number of samples in each source category, the model’s specificity may have been reduced, leading to a less granulated attribution analysis. Despite the model’s accuracy, a considerable number of cases were assigned to a specific source with only a low or moderate probability level. This could indicate that *Campylobacter* strains are widely dispersed across sources, making it more difficult to distinguish each strain from each other. Increasing the size of the *k*-mers used in the analysis could potentially improve the model’s discriminatory by capturing more of the genomic content, but this approach presents different computational challenges.

As the *k*-mer length increases, the number of possible combinations for each of the four nucleotides grows exponentially, which leads to computational constraints. Longer *k*-mers also reduce the availability of information on their frequencies across samples, as they can become too specific. On the other hand, sequencing errors are much more likely to confound the analysis when using short *k*-mers. This is why using high-quality genomes is important, because it minimizes biases in recognizing genetic patterns across individual sources. The key to effective use of *k*-mers is to capture trends in their frequencies without losing critical information. Based on these considerations, 9-mers were selected as the optimal length for this study.

All these results highlight the importance of having a complete and representative sampling strategy, where household animals and wildlife are also included in the monitoring system, while maintaining a high number of samples. Absence of data for a particular source in a specific country does not necessarily imply that the source is not a potential cause of disease. For instance, no cases of human campylobacteriosis were found to be attributed to wild birds, despite evidence from other countries indicating that wild birds can be a reservoir for *Campylobacter* (Cody et al., 2015). Gaps like this in the available data can influence source attribution model outcomes, potentially leading to an underestimation of important sources. If the monitoring system focuses exclusively on the production chain, the source attribution models will only identify cases linked to sources within that chain. However, *Campylobacter* is also transmitted through environmental reservoirs, such as wildlife and household animals (Zenebe et al., 2020; Mughini-Gras et al., 2021). While these reservoirs may not account for the largest proportion of infections, their inclusion in monitoring systems is essential for a comprehensive understanding of *Campylobacter* transmission dynamics. By incorporating data from household animals and wildlife, the model will gain a more comprehensive base of potential reservoirs, reducing the risk of underestimating their role in disease transmission. This could also improve model performance, since the model will be able to accurately attribute cases to their original source. This is especially important for implementing effective control strategies, since overlooking these sources could underestimate the effect of implemented action plans. Therefore, a more representative sampling strategy that includes household animals and wildlife is essential to better inform public health interventions.

The model also found that the majority of cases were attributed to livestock (broilers and cattle). Other studies have also found livestock to be the largest reservoir of *Campylobacter* infections, but it is likely that predictions are skewed toward these sources simply because of their overrepresentation. Overestimation can, therefore, also happen, making it even more evident that sampling should be standardized in all aspects. To clarify this, when further exploring the impact of sample size on source attribution, it becomes clear that more samples from a specific source like broilers not only introduces more genetic variation in the samples included in the model but also increases the probability that a random human case of campylobacteriosis is allocated to that source. Conversely, a source with fewer samples will have a weaker ability to detect rare types in a reservoir that is sparsely sampled. This discrepancy is critical for understanding the concepts of sensitivity and specificity within this context. For example, the Netherlands showcased a well-distributed sample collection across various sources, which results in more balanced outcomes in the source attribution models. This approach helps in preventing the misallocation of cases to sources that are overrepresented in the dataset, emphasizing the necessity for an evenly distributed number of samples from each potential source to avoid biased results. Legislation should be more focused on implementing more samples in the regulatory sampling strategy for source-attribution studies in the future. The quantity of samples and the diversity of sources from where they are collected play an important role when using source attribution models to predict human campylobacteriosis cases across multiple countries. To improve future models and the effectiveness of surveillance programs, efforts should be made to ensure that multiple relevant sources are covered, including those that might not have been recognized yet as large contributors. This can be achieved by implementing a One Health strategy, combining the analysis of human, animal, and environmental samples. The sampling strategy largely depends on national priorities and available resources, as each country must determine the most feasible approach for its monitoring system. However, our study demonstrates that adopting a broader and more representative sampling strategy could significantly enhance surveillance by capturing a wider range of potential sources. To minimize bias, we recommend designing sampling plans that aim for a more balanced distribution of samples across sources. Over-representing one source, such as broilers, may not necessarily provide additional valuable information and can skew the model’s attribution outcomes. A more equitable sampling strategy would help ensure that undersampled but potentially important reservoirs are adequately captured, improving the overall accuracy and utility of surveillance and source attribution efforts.

## Data Availability

Publicly available datasets were analyzed in this study. This data can be found at: The data that support the findings of this study are all available in the European Nucleotide Archive (ENA) under accession numbers PRJEB58624 (SSI), PRJEB59243 (DTU), PRJNA854907 (DAFM), PRJEB38253 (RIVM), PRJEB58185 (PIWET), PRJEB57556 (VISAVET-UCM), PRJNA688841, PRJNA665357 (TEAGASC), PRJEB46750 (INIAV/INSA), PRJNA645794, PRJNA534408 (INIAV), PRJEB75207 (ANSES), PRJEB60264 and PRJEB34530 (SVA). Additional data was provided by the public health laboratory of Ireland. Data is available upon reasonable request.

## Footnotes

^1^ https://onehealthejp.eu/jrp-discover
