# Supplementary material for: Source attribution of human Campylobacter infection: a multi-country model in the European Union
Source: Front Microbiol. 2025 Feb 5;16:1519189. doi: 10.3389/fmicb.2025.1519189 (PMC11835883; doi:10.3389/fmicb.2025.1519189)
Supplement: Supplementary file 1 [file Data_Sheet_1.docx]

Supplementary Material

Source attribution of human Campylobacter infection: A multi-country model in the European Union

Thystrup et al.

Contents

[Supplementary Table S1. Valid accuracy for the country-specific attribution models. 2](#_Toc164772858)

[Supplementary Figure S1: Cumulative probability of all human cases of campylobacteriosis in Spain attributed by the individual country model (n=128). Each bar represents one human case, with the height of the bar representing the probability of the case to originate from a specific source. 4](#_Toc164772859)

[Supplementary Figure S2: Cumulative probability of all human cases of campylobacteriosis in Ireland attributed by the individual country model (n=267). Each bar represents one human case, with the height of the bar representing the probability of the case to originate from a specific source. 5](#_Toc164772860)

[Supplementary Figure S3: Cumulative probability of all human cases of campylobacteriosis in Poland attributed by the individual country model (n=8). Each bar represents one human case, with the height of the bar representing the probability of the case to originate from a specific source. 6](#_Toc164772861)

[Supplementary Figure S4: Cumulative probability of all human cases of campylobacteriosis in the Netherlands attributed by the individual country model (n=280). Each bar represents one human case, with the height of the bar representing the probability of the case to originate from a specific source. 7](#_Toc164772862)

[Supplementary Figure S5: Cumulative probability of all human cases of campylobacteriosis in Portugal attributed by the individual country model (n=379). Each bar represents one human case, with the height of the bar representing the probability of the case to originate from a specific source. 8](#_Toc164772863)

[Supplementary Figure S6: Cumulative probability of all human cases of campylobacteriosis in Denmark attributed by the individual country model (n=1,558). Each bar represents one human case, with the height of the bar representing the probability of the case to originate from a specific source. 9](#_Toc164772864)

[Supplementary Table S2: Valid accuracy and kappa value for the multi-country models using the baseline dataset and the down-sampled dataset. 10](#_Toc164772865)

[Supplementary Table S3: Sensitivity, specificity, and balanced accuracy for the predicted sources by the logit-boost machine learning model, using the down-sampled data. 11](#_Toc164772866)

[Supplementary Table S4: Sensitivity, specificity, and balanced accuracy for the predicted sources by the random forest machine learning model, using the up-sampled data. 13](#_Toc164772867)

[Supplementary Table S5: Number of human campylobacteriosis cases attributed to each source using the baseline random forest model, stratified by country. Only cases with a ≥70% probability of being attributed to a source are shown. 15](#_Toc164772868)

[Supplementary Table S6: Number of human campylobacteriosis cases attributed to each source using the down-sampled logit boost model, stratified by country. Only cases with a ≥70% probability of being attributed to a source are shown. 16](#_Toc164772869)

# **Supplementary Table S1**. Valid accuracy for the country-specific attribution models.

|  | Model selection | | Model construction | | |
| --- | --- | --- | --- | --- | --- |
|  | **Random forest** | **Logit boost** | **Selected algorithm** | **Valid accuracy** | **Kappa** |
| Denmark | 0.788 | 0.784 | Random forest | 0.796 | 0.555 |
| Ireland | 0.925 | 0.934 | Logit boost | 0.944 | 0.483 |
| Netherlands | 0.610 | 0.598 | Random forest | 0.613 | 0.536 |
| Poland | 0.905 | 0.959 | Logit boost | 0.945 | 0.869 |
| Portugal | 0.691 | 0.673 | Random forest | 0.673 | 0.526 |
| Spain | 0.507 | 0.540 | Logit boost | 0.540 | 0.327 |

# **Supplementary Figure S1:** Cumulative probability of all human cases of campylobacteriosis in Spain attributed by the individual country model (n=128). Each bar represents one human case, with the height of the bar representing the probability of the case to originate from a specific source.


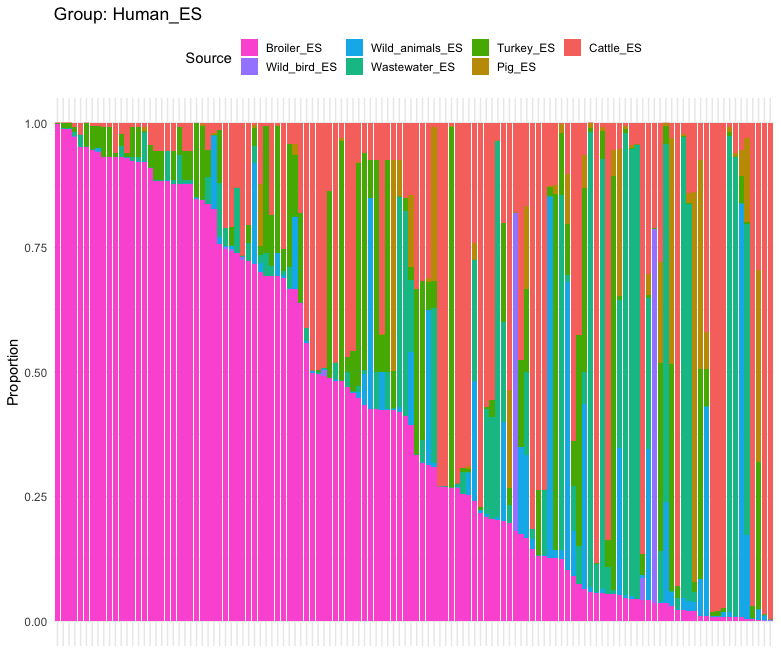


# **Supplementary Figure S2:** Cumulative probability of all human cases of campylobacteriosis in Ireland attributed by the individual country model (n=267). Each bar represents one human case, with the height of the bar representing the probability of the case to originate from a specific source.


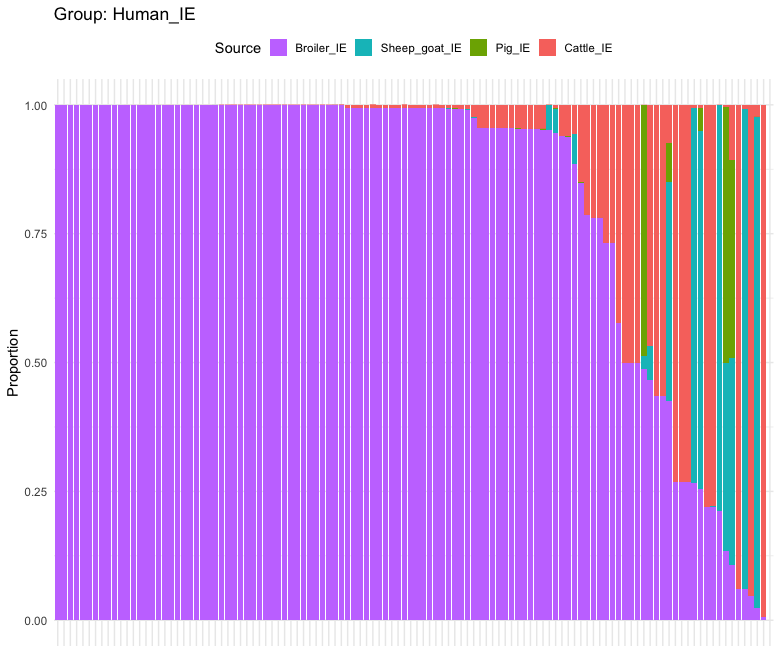


# **Supplementary Figure S3**: Cumulative probability of all human cases of campylobacteriosis in Poland attributed by the individual country model (n=8). Each bar represents one human case, with the height of the bar representing the probability of the case to originate from a specific source.


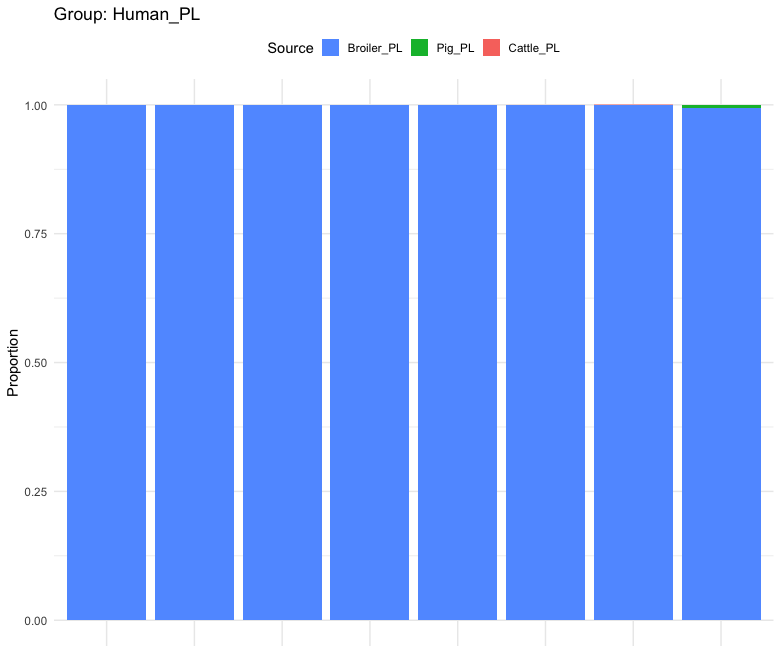


# **Supplementary Figure S4:** Cumulative probability of all human cases of campylobacteriosis in the Netherlands attributed by the individual country model (n=280). Each bar represents one human case, with the height of the bar representing the probability of the case to originate from a specific source.


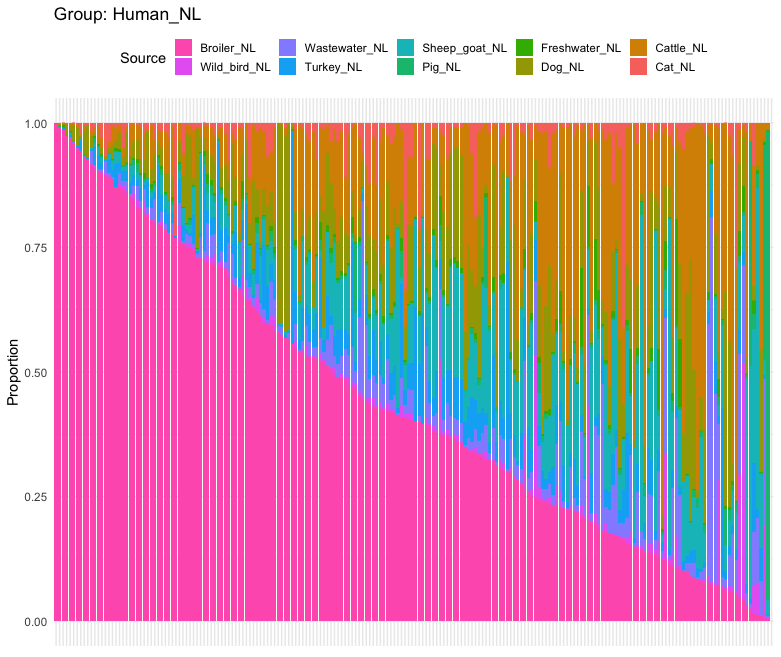


# **Supplementary Figure S5**: Cumulative probability of all human cases of campylobacteriosis in Portugal attributed by the individual country model (n=379). Each bar represents one human case, with the height of the bar representing the probability of the case to originate from a specific source.


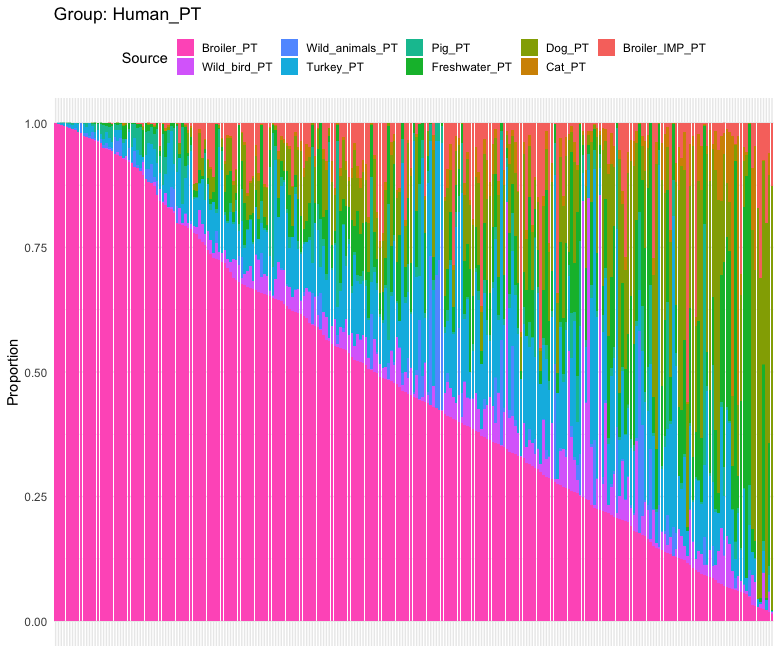


# **Supplementary Figure S6:** Cumulative probability of all human cases of campylobacteriosis in Denmark attributed by the individual country model (n=1,558). Each bar represents one human case, with the height of the bar representing the probability of the case to originate from a specific source.

*
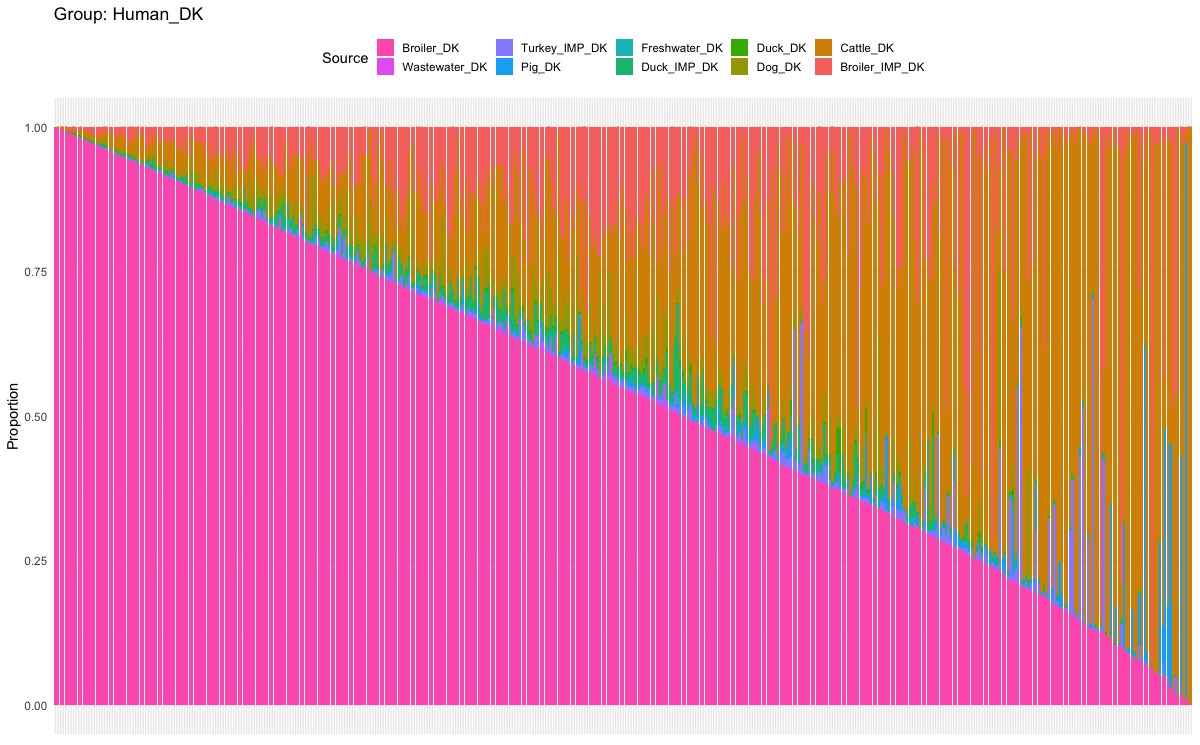
*

# **Supplementary Table S2:** Valid accuracy and kappa value for the multi-country models using the baseline dataset and the down-sampled dataset.

|  | Valid accuracy (95% CI) | Kappa |
| --- | --- | --- |
| Baseline model | 0.675 (0.65-0.699)* | 0.653 |
| Down-sampled model | 0.646 (0.622-0.685** | 0.642 |

*Constructed using random forest

** Constructed using logit-boost

# **Supplementary Table S3**: Sensitivity, specificity, and balanced accuracy for the predicted sources by the logit-boost machine learning model, using the down-sampled data.

|  | Broiler_DK | Broiler_ES | Broiler_FR | Broiler_IE | Broiler_IMP_DK | Broiler_IMP_PT | Broiler_NL | Broiler_PL |
| --- | --- | --- | --- | --- | --- | --- | --- | --- |
| Sensitivity | 0.52778 | 0.2 | 0 | 0.78261 | 0.272727 | 1.0 | 0.36735 | 0.6667 |
| Specificity | 0.94174 | 0.989922 | 0.998889 | 0.98223 | 0.971942 | 0.99888 | 0.97685 | 0.98398 |
| Balanced accuracy | 0.73476 | 0.594961 | 0.499444 | 0.88242 | 0.622334 | 0.99944 | 0.67210 | 0.82532 |
|  | Broiler_PT | Broiler_SE | Cat_FR | Cat_NL | Cat_PT | Cattle_DK | Cattle_ES | Cattle_FR |
| Sensitivity | 0.65000 | 0.272727 | 1.0 | 0.76923 | 1.0 | 0.6 | 0 | 0.166667 |
| Specificity | 0.98544 | 1.0 | 1.0 | 0.99556 | 1.0 | 0.97938 | 0.996707 | 0.995590 |
| Balanced accuracy | 0.81772 | 0.636364 | 1.0 | 0.88239 | 1.0 | 0.78969 | 0.498353 | 0.581128 |
|  | Cattle_IE | Cattle_NL | Cattle_PL | Dog_DK | Dog_FR | Dog_NL | Dog_PT | Duck_DK |
| Sensitivity | 0.888889 | 0.296296 | 1.0 | 0.75 | 0.68750 | 1.0 | 0.90909 | 1.0 |
| Specificity | 0.995575 | 0.974041 | 1.0 | 0.996685 | 0.99889 | 0.994401 | 0.99889 | 1.0 |
| Balanced accuracy | 0.942232 | 0.635168 | 1.0 | 0.873343 | 0.84319 | 0.5472 | 0.95399 | 1.0 |
|  | Duck_IMP_DK | Freshwater_DK | Freshwater_FR | Freshwater_NL | Freshwater_PT | Pig_DK | Pig_ES | Pig_FR |
| Sensitivity | 0.625 | 1.0 | 0.714286 | 0.071429 | 0.818182 | 1.0 | 1.0 | 1.0 |
| Specificity | 0.99779 | 1.0 | 0.996689 | 0.991101 | 0.997783 | 1.0 | 0.99666 | 0.99666 |
| Balanced accuracy | 0.811395 | 1.0 | 0.855487 | 0.531265 | 0.907982 | 1.0 | 0.99833 | 0.99833 |
|  | Pig_IE | Pig_NL | Pig_PL | Pig_PT | Sheep_goat_IE | Sheep_goat_NL | Turkey_ES | Turkey_IMP_DK |
| Sensitivity | 1.0 | 0.52174 | 0.428571 | 1.0 | 1.0 | 0.461538 | 0.6 | 1.0 |
| Specificity | 1.0 | 0.99438 | 0.992214 | 1.0 | 0.99889 | 0.975556 | 0.988987 | 0.997788 |
| Balanced accuracy | 1.0 | 0.75806 | 0.710392 | 1.0 | 0.99944 | 0.718547 | 0.794493 | 0.998894 |
|  | Turkey_NL | Turkey_PT | Wastewater_DK | Wastewater_ES | Wastewater_NL | Wild_animals_ES | Wild_animals_FR | Wild_animals_PT |
| Sensitivity | 0 | 0.615385 | 1.0 | 0.888889 | 0.52174 | 0.857143 | 1.0 | 1.0 |
| Specificity | 0.992291 | 1.0 | 1.0 | 1.0 | 0.98385 | 0.996689 | 1.0 | 1.0 |
| Balanced accuracy | 0.496145 | 0.807692 | 1.0 | 1.0 | 0.75280 | 0.926916 | 1.0 | 1.0 |
|  | Wild_bird_ES | Wild_bird_FR | Wild_bird_NL | Wild_bird_PT | Wild_bird_SE |  |  |  |
| Sensitivity | 0.857143 | 1.0 | 0.363636 | 1.0 | 0.75 |  |  |  |
| Specificity | 0.996689 | 0.9967 | 0.992239 | 1.0 | 0.998895 |  |  |  |
| Balanced accuracy | 0.926916 | 0.99835 | 0.677938 | 1.0 | 0.874448 |  |  |  |

# **Supplementary Table S4:** Sensitivity, specificity, and balanced accuracy for the predicted sources by the random forest machine learning model, using the up-sampled data.

|  | Broiler_DK | Broiler_ES | Broiler_FR | Broiler_IE | Broiler_IMP_DK | Broiler_IMP_PT | Broiler_NL | Broiler_PL |
| --- | --- | --- | --- | --- | --- | --- | --- | --- |
| Sensitivity | 0.8142 | 0.214286 | 0.090909 | 0.78481 | 0.413793 | 1.0 | 0.3494 | 0.77778 |
| Specificity | 0.8771 | 0.991603 | 0.998606 | 0.97678 | 0.986695 | 0.999307 | 0.96652 | 0.98938 |
| Balanced accuracy | 0.8457 | 0.602944 | 0.544758 | 0.88079 | 0.700244 | 0.999654 | 0.65796 | 0.88358 |
|  | Broiler_PT | Broiler_SE | Cat_FR | Cat_NL | Cat_PT | Cattle_DK | Cattle_ES | Cattle_FR |
| Sensitivity | 0.307692 | 0.5 | 1.0 | 0.89474 | 1.0 | 0.55263 | 0.266667 | 0.333333 |
| Specificity | 0.998602 | 0.997228 | 0.99861 | 1.0 | 0.999309 | 0.97466 | 0.998613 | 0.998610 |
| Balanced accuracy | 0.653147 | 0.748614 | 0.99931 | 0.94737 | 0.999654 | 0.76364 | 0.632640 | 0.665972 |
|  | Cattle_IE | Cattle_NL | Cattle_PL | Dog_DK | Dog_FR | Dog_NL | Dog_PT | Duck_DK |
| Sensitivity | 0.85 | 0.26471 | 1.0 | 0.928571 | 0.769231 | 0.217391 | 0.769231 | 1.0 |
| Specificity | 0.99861 | 0.98128 | 0.999308 | 1.0 | 0.997922 | 0.993724 | 0.993767 | 1.0 |
| Balanced accuracy | 0.92430 | 0.62299 | 0.999654 | 0.964286 | 0.883577 | 0.605558 | 0.881499 | 1.0 |
|  | Duck_IMP_DK | Freshwater_DK | Freshwater_FR | Freshwater_NL | Freshwater_PT | Pig_DK | Pig_ES | Pig_FR |
| Sensitivity | 0.9375 | 1.0 | 0.6875 | 0.0 | 1.0 | 0.916667 | 1.0 | 1.0 |
| Specificity | 1.0 | 0.99931 | 0.999306 | 0.996533 | 0.999307 | 1.0 | 1.0 | 0.997922 |
| Balanced accuracy | 0.96875 | 0.99965 | 0.843403 | 0.498266 | 0.999654 | 0.958333 | 1.0 | 0.998961 |
|  | Pig_IE | Pig_NL | Pig_PL | Pig_PT | Sheep_goat_IE | Sheep_goat_NL | Turkey_ES | Turkey_IMP_DK |
| Sensitivity | 1.0 | 0.87805 | 0.44118 | 1.0 | 1.0 | 0.28125 | 0.611111 | 1.0 |
| Specificity | 1.0 | 0.98941 | 0.99649 | 0.999311 | 0.997928 | 0.992281 | 0.997915 | 0.99791 |
| Balanced accuracy | 1.0 | 0.93373 | 0.71883 | 0.999655 | 0.998964 | 0.636765 | 0.804513 | 0.99896 |
|  | Turkey_NL | Turkey_PT | Wastewater_DK | Wastewater_ES | Wastewater_NL | Wild_animals_ES | Wild_animals_FR | Wild_animals_PT |
| Sensitivity | 0.416667 | 1.0 | 1.0 | 1.0 | 0.68421 | 1.0 | 1.0 | 1.0 |
| Specificity | 0.997924 | 0.999308 | 0.99722 | 0.999308 | 0.98357 | 0.995845 | 1.0 | 0.998616 |
| Balanced accuracy | 0.707295 | 0.999654 | 0.99861 | 0.999654 | 0.83389 | 0.997922 | 1.0 | 0.999308 |
|  | Wild_bird_ES | Wild_bird_FR | Wild_bird_NL | Wild_bird_PT | Wild_bird_SE |  |  |  |
| Sensitivity | 1.0 | 0.6875 | 0.263158 | 1.0 | 0.705882 |  |  |  |
| Specificity | 1.0 | 0.999306 | 0.997218 | 1.0 | 0.997222 |  |  |  |
| Balanced accuracy | 1.0 | 0.843403 | 0.630188 | 1.0 | 0.851552 |  |  |  |

# **Supplementary Table S5:** Number of human campylobacteriosis cases attributed to each source using the baseline random forest model, stratified by country. Only cases with a ≥70% probability of being attributed to a source are shown.

|  |  | Cases (DK) | Cases (NL) | Cases (PT) | Cases (IE) | Cases (PL) |
| --- | --- | --- | --- | --- | --- | --- |
| Denmark (DK) | Broiler | 199 | 3 | 3 | 3 | - |
|  | Broiler (imported) | 6 | - | - | 1 | - |
|  | Cattle | 19 | - | - | - | - |
|  | Pig | 1 | - | - | - | - |
| Netherlands (NL) | Broiler | 2 | 5 | - | - | - |
| Portugal (PT) | Broiler | - | - | 5 | - | - |
| Ireland (IE) | Broiler | - | - | - | 12 | - |
| Poland (PL) | Broiler | 2 | - | 1 | - | 7 |
| Spain (ES) | Broiler | - | - | - | 1 | - |
| France (FR) | Dog | 1 | - | - | - | - |
|  | Cattle | 1 | - | - | - | - |
| Total # of cases |  | 232 | 8 | 9 | 17 | 7 |

# **Supplementary Table S6:** Number of human campylobacteriosis cases attributed to each source using the down-sampled logit boost model, stratified by country. Only cases with a ≥70% probability of being attributed to a source are shown.

|  |  | Cases (DK) | Cases (NL) | Cases (PT) | Cases (IE) | Cases (PL) | Cases (ES) |
| --- | --- | --- | --- | --- | --- | --- | --- |
| Denmark (DK) | Broiler | 226 | 26 | 16 | 8 | - | 3 |
|  | Broiler (imported) | 7 | 1 | 1 | 2 | - | - |
|  | Cattle | 54 | 3 | - | 4 | - | - |
|  | Pig | 3 | - | - | - | - | - |
|  | Dog | 1 | - | - | - | - | - |
|  | Duck (imported) | 2 | - | - | 1 | - | - |
| Netherlands (NL) | Broiler | 23 | 22 | 2 | 1 | - | - |
|  | Cattle | 4 | 6 | - | 2 | - | 1 |
|  | Dog | 4 | 2 | 1 | - | - | - |
|  | Pig | 1 | - | 1 | - | - | - |
|  | Sheep/goat | 5 | 1 | - | - | - | - |
|  | Turkey | 2 | - | - | - | - | - |
|  | Wastewater | 2 | 1 | 1 | - | - | - |
|  | Freshwater | - | - | - | 1 | - | - |
| Portugal (PT) | Broiler | 2 | - | 11 | - | - | - |
|  | Dog | 1 | - | 1 | - | - | - |
|  | Turkey | - | - | 1 | - | - | - |
|  | Wild birds | - | - | 7 | - | - | - |
| Ireland (IE) | Broiler | 4 | 1 | - | 8 | - | 1 |
|  | Cattle | 1 | - | - | 1 | - | - |
|  | Sheep/goat | - | - | - | - | - | 1 |
| Poland (PL) | Broiler | 6 | 2 | 2 | - | 8 | - |
|  | Pig | 2 | - | - | - | - | - |
| Spain (ES) | Broiler | 3 | - | 2 | 2 | - | 8 |
|  | Cattle | 2 | - | - | 1 | - | - |
|  | Turkey | - | 1 | - | - | - | - |
| France (FR) | Broiler | 3 | - | - | - | - | - |
|  | Dog | 2 | - | - | - | - | - |
|  | Wild birds | 2 | - | - | - | - | - |
|  | Cattle | - | 1 | - | - | - | - |
| Sweden | Broiler | 3 | 1 | - | 1 | - | - |
| Total # of cases |  | 365 | 68 | 46 | 34 | 8 | 14 |
